# A Case of Isolated Epstein-Barr Virus Hepatitis With Jaundice

**DOI:** 10.7759/cureus.111527

**Published:** 2026-06-26

**Authors:** Reem M Al Makari, Zyad Mroue, Ghenwa K El Dakdoukii

**Affiliations:** 1 Infectious Disease, Hammoud Hospital University Medical Center, Saida, LBN; 2 Infectious Disease, Beirut Arab University, Beirut, LBN; 3 Internal Medicine, Hammoud Hospital University Medical Center, Saida, LBN

**Keywords:** acute acalculous cholecystitis (aac), acute cholangitis, bacteria and viruses causing jaundice, ebv-associated hepatitis, epstein-barr virus (ebv)

## Abstract

Epstein-Barr virus (EBV), presenting as infectious mononucleosis (IM), affects almost all people by age 20. It has several systemic manifestations that are associated with pharyngitis and fever. We present a rare presentation of EBV infection manifesting as hepatitis and jaundice without prior IM symptoms. A 46-year-old female presented with abdominal pain and jaundice. Laboratory findings showed elevated liver enzymes and bilirubin. There was no evidence of other diseases on laboratory findings, imaging, or symptoms. EBV IgM was the only laboratory-positive value. The patient continued symptomatic treatment and fully recovered within about 10 months. EBV manifesting as isolated hepatitis occurs rarely in the population. Cases of hepatitis not preceded by IM have not been reported previously.

## Introduction

Epstein-Barr virus (EBV) or human herpesvirus 4 is a gammaherpesvirus that infects individuals by age 20. Primary infection is asymptomatic in children, but in adolescents and adults, it manifests as infectious mononucleosis (IM) with fever, pharyngitis, lymphadenopathy, and splenomegaly. Hepatic manifestation is frequent in mononucleosis caused by EBV and typically represents mild and transient elevation of aminotransferases two- to three-fold above the normal range [1-3].

Isolated hepatitis due to EBV is unusual, and EBV results in rare forms of acute and chronic hepatitis [3,4]. Although generally self-limiting, it is challenging to diagnose when classic mononucleosis is not present. Isolated EBV hepatitis presenting without the classical manifestations of IM is uncommon and has been reported sporadically in the literature. Here, we present a case of isolated hepatitis and jaundice due to EBV in an otherwise fit middle-aged woman.

## Case presentation

A 46-year-old previously healthy female presented to the emergency department with a two-day history of nausea, dysuria, abdominal pain, and jaundice. She did not have a history of recent travel, alcohol consumption, herbal medication, sick contacts, or previous liver disease. The patient had no fever or symptoms suspicious of IM. On admission, she was oriented, alert, and afebrile (36.7°C), with normal vital signs and saturation. On physical examination, she was icteric, with mild epigastric tenderness, positive Murphy sign, but no abdominal distension or hepatosplenomegaly.

Laboratory investigations (Table 1) revealed markedly elevated aminotransferases, hyperbilirubinemia, raised alkaline phosphatase and gamma-glutamyl transferase, and mildly elevated C-reactive protein. Complete blood count was within normal limits except for borderline thrombocytopenia (platelets 138 × 10³/µL). Serological testing for hepatitis A, B, and C was negative. Hepatitis D and E infections were less likely given the local epidemiology and absence of specific risk factors, although testing was unavailable.

**Table 1:** Laboratory results upon admission. WBC = white blood cells; SGOT = serum glutamic-oxaloacetic transaminase; SGPT = serum glutamic-pyruvic transaminase; ALP = alkaline phosphatase; GGT = gamma-glutamyl transferase; T/D = total/direct; CRP = C-reactive protien; INR = international normalized ratio

Parameter	Result	Normal range
WBC	5.61 × 10³/µL	4–11 × 10³/µL
Neutrophils	64%	40–65%
Hemoglobin	12.6 g/dL	12–16 g/dL
Hematocrit	37.5%	37–46%
Platelets	138 × 10³/µL	150–400 × 10³/µL
Creatinine	0.7 mg/dL	0.55–1.10 mg/dL
SGOT	1,143 U/L	0–30 U/L
SGPT	1,012 U/L	0–33 U/L
ALP	132 U/L	30–120 IU/L
GGT	204 U/L	0–37 U/L
Bilirubin (T/D)	8.1/5.7 mg/dL	0.3–1.2 mg/dL/0–0.5 mg/dL
CRP	12 mg/L	<5 mg/L
INR	1.61	1.00–1.17

IgG level was 16.4 g/L, antinuclear antibody was borderline positive, and antismooth muscle, antimitochondrial, anti-SSA, and anti-SSB antibodies were all negative, resulting in a score of 4 for autoimmune hepatitis criteria, which excluded the diagnosis. Cytomegalovirus IgM was negative, and EBV IgM (anti-VCA) was positive (1.891, reactive).

**Figure 1:**
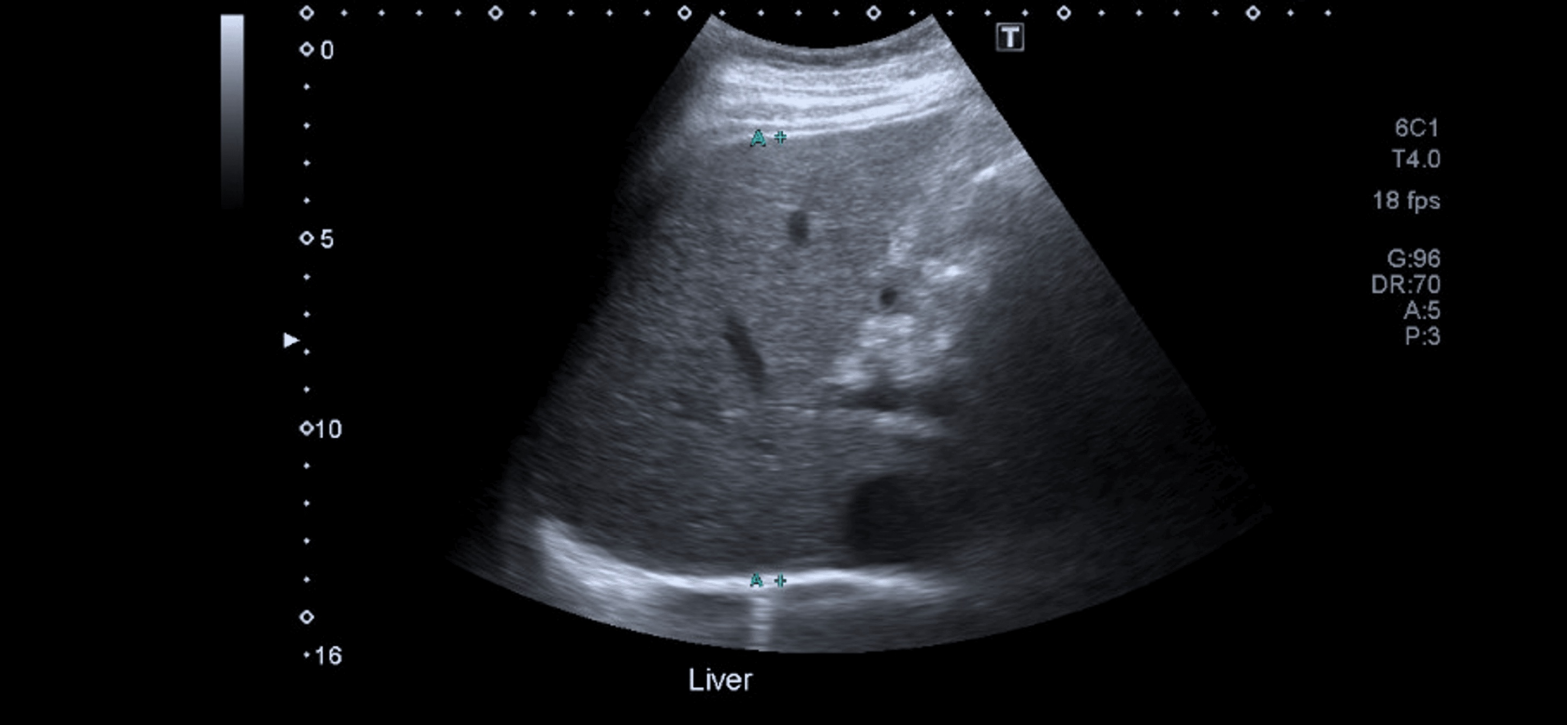
Ultrasound showing the liver.

**Figure 2:**
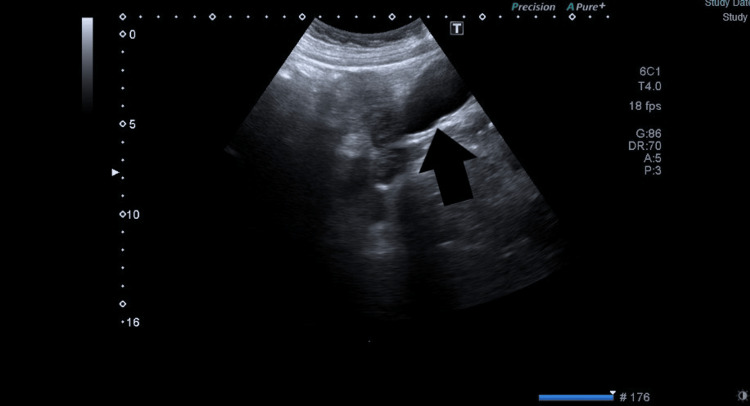
Ultrasound showing the gallbladder.

**Figure 3:**
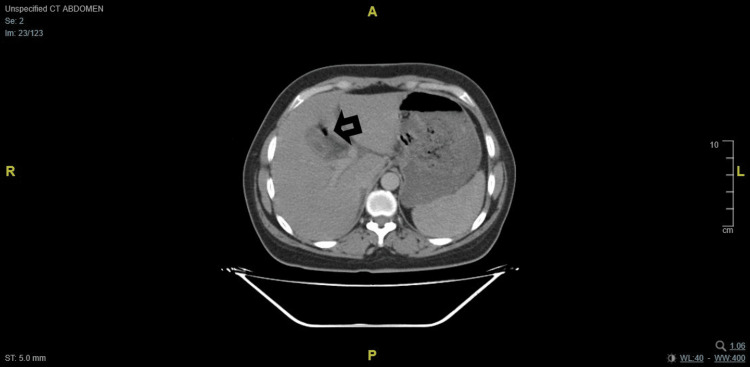
CT showing a distended gallbladder.

Abdominal ultrasound (Figures 1, 2) and abdominal CT (Figure 3) demonstrated gallbladder wall thickening and pericholecystic fluid, with an absence of gallstones or biliary obstruction, favoring reactive gallbladder inflammation secondary to hepatitis rather than primary acalculous cholecystitis.

Liver enzymes were maximal at presentation and gradually decreased during and after hospitalization (Table 2).

**Table 2:** Liver enzymes trend during hospitalization and follow-up. SGOT = serum glutamic-oxaloacetic transaminase; SGPT = serum glutamic-pyruvic transaminase; T/D = total/direct; INR = international normalized ratio

Time	Normal range	On admission	Day 6	Day 9	Month 1	Month 3	Month 10
SGOT (U/L)	0–30	1,143	878	220	121	75	29
SGPT (U/L)	0–33	1,012	771	328	126	86	21
Bilirubin (T/D) (mg/dL)	0.3–1.2/0–0.5	8.1/5.7	15.8/10.7	15.8/11.4	5.6/2.6	1.1/0.6	-
INR	1–1.17	1.61	-	-	1.18	-	-

She was discharged on day 14. One month later, liver enzymes improved, and the ultrasound showed normalization. Liver function tests were normal at 10 months.

## Discussion

EBV infection is prevalent, with more than 90% seroprevalence among adults [1]. Although hepatic disease occurs in IM, clinically apparent hepatitis with jaundice occurs infrequently (<1% of cases) [2-5]. Our patient had jaundice and abdominal pain without features of mononucleosis, demonstrating the diagnostic dilemma of isolated EBV hepatitis.

The pathogenetic mechanism differs from that of hepatotropic viruses (hepatitis B and C), which infect hepatocytes themselves. EBV does not infect hepatocytes, but infected CD8⁺ T lymphocytes are found in the liver and release cytokines such as interferon-gamma, tumor necrosis factor-α, and Fas ligand, which are implicated in hepatocellular injury [6].

Diagnosis relies on EBV-specific serology. Anti-VCA IgM is positive in acute infection, while VCA IgG and EBNA-1 IgG are useful in the discrimination of acute, past, or reactivated infection [7-9]. Monospot is quicker but less sensitive [5]. Clinical presentation and laboratory findings help diagnose EBV hepatitis [2]. Diagnosis was confirmed in this case based on a positive anti-VCA IgM after excluding other hepatotropic viruses and autoimmune hepatitis. Although the patient had borderline antinuclear antibody, the absence of other autoimmune markers and the clinical course made autoimmune hepatitis unlikely.

A limitation of this report is that the diagnosis of acute EBV infection was based primarily on positive anti-VCA IgM serology. Additional confirmatory testing, such as EBV viral load by polymerase chain reaction, VCA IgG, and EBNA antibody testing, was not available. Nevertheless, the exclusion of common viral and autoimmune causes of hepatitis, together with the patient’s clinical presentation and spontaneous biochemical recovery, supports acute EBV infection as the most likely etiology.

Treatment of EBV hepatitis is supportive. Antiviral therapy with acyclovir, ganciclovir, interferon, or interleukin-2 has not proven consistently effective in clinical response [10]. Our patient was treated with supportive care and antibiotics for secondary acalculous cholecystitis after she developed borderline blood pressure, elevation of C-reactive protein, and an ill-appearing presentation raising suspicion of secondary superimposed bacterial infection.

An unusual feature of this case was the prolonged biochemical recovery, with complete normalization occurring only after 10 months. EBV-related liver injury is usually self-limiting and is accompanied by a mild-to-moderate increase in liver enzymes, starting in the first week, peaking in the second, and returning to normal in the third [2,3]. These delayed recoveries have been rarely documented [8,9]. Delayed recovery serves to underscore the variability in the natural history of EBV hepatitis and the requirement for long-term follow-up to confirm recovery.

Our case shares features with previous reports of isolated, mononucleosis-absent EBV hepatitis in immunocompetent hosts, such as that described by Moniri et al. [8], in which systemic features were absent, and outcomes were favorable. However, it differed from the study reported by Patel et al. [11], in which the patient lacked systemic mononucleosis manifestations. However, in contrast to the rapid biochemical improvement and normalization within weeks to six months described in previous reports, such as the encephalitis-associated, mild hepatitis described by Elhadidy et al. [9], our patient had severe cholestatic jaundice with an unusually prolonged recovery (10 months) despite early clinical improvement. It illustrates the wide temporal spectrum of EBV-mediated liver injury and supports extended laboratory follow-up to confirm complete biochemical resolution following cryptogenic cholestatic hepatitis.

## Conclusions

Isolated EBV hepatitis must be a differential diagnosis in adult patients with acute hepatitis and jaundice when common viral and autoimmune causes have been excluded. Diagnosis is confirmed by EBV serology, and therapy is mainly supportive. Although EBV hepatitis is usually self-limiting, biochemical recovery may be substantially delayed in some patients, emphasizing the importance of longitudinal follow-up.
